# Genomic SNP array as a gold standard for prenatal diagnosis of foetal ultrasound abnormalities

**DOI:** 10.1186/1755-8166-5-14

**Published:** 2012-03-13

**Authors:** Malgorzata I Srebniak, Marjan Boter, Gretel O Oudesluijs, Titia Cohen-Overbeek, Lutgarde CP Govaerts, Karin EM Diderich, Renske Oegema, Maarten FCM Knapen, Ingrid MBH van de Laar, Marieke Joosten, Diane Van Opstal, Robert-Jan H Galjaard

**Affiliations:** 1 Department of Clinical Genetics, Erasmus MC, Rotterdam, the Netherlands; 2 Department of Obstetrics and Gynaecology, Division of Obstetrics and Prenatal Medicine, Erasmus MC, Rotterdam, the Netherlands

**Keywords:** Genomic SNP array, Prenatal diagnosis, Foetal abnormalities, Submicroscopic aberrations

## Abstract

**Background:**

We have investigated whether replacing conventional karyotyping by SNP array analysis in cases of foetal ultrasound abnormalities would increase the diagnostic yield and speed of prenatal diagnosis in clinical practice.

**Findings/results:**

From May 2009 till June 2011 we performed HumanCytoSNP-12 array (HCS) (http://www.Illumina.com) analysis in 207 cases of foetal structural abnormalities. HCS allows detecting unbalanced genomic abnormalities with a resolution of about 150/200 kb. All cases were selected by a clinical geneticist after excluding the most common aneuploidies by RAD (rapid aneuploidy detection). Pre-test genetic counselling was offered in all cases.

In 24/207 (11,6%) foetuses a clinically relevant genetic abnormality was detected. Only 8/24 abnormalities would have been detected if only routine karyotyping was performed. Submicroscopic abnormalities were found in 16/207 (7,7%) cases. The array results were achieved within 1-2 weeks after amniocentesis.

**Conclusions:**

Prenatal SNP array testing is faster than karyotyping and allows detecting much smaller aberrations (~0.15 Mb) in addition to the microscopic unbalanced chromosome abnormalities detectable with karyotyping (~ > 5 Mb). Since karyotyping would have missed 66% (16/24) of genomic abnormalities in our cohort, we propose to perform genomic high resolution array testing assisted by pre-test counselling as a primary prenatal diagnostic test in cases of foetal ultrasound abnormalities.

## Findings

Array based diagnosis of foetal unbalanced chromosome abnormalities has been successfully employed on prenatal material [1,2]. However, the standard cytogenetic prenatal diagnosis often still includes time-consuming karyotyping followed by targeted FISH or MLPA analysis when a submicroscopic abnormality is suspected. Although foetal anomalies are a strong indication of a genetic abnormality, chromosome analysis can only detect aberrations in about one fifth of cases [3]. In 1999-2010 we tested 3076 cases of foetuses with abnormal ultrasound findings and 21,7% (665) had an abnormal karyotype (including triploidy, trisomy 13, 18, 21 and aneuploidies of X and Y chromosome).

Prenatal genetic diagnosis after ultrasound detection of foetal abnormalities requires a fast diagnostic technique. A technique with higher resolution than karyotyping is preferable to avoid the need of subsequent microdeletion/microduplication testing. Implementing rapid microarray technology in routine prenatal diagnosis will allow the detection of unbalanced chromosomal abnormalities in a shorter period of time with much better resolution than conventional karyotyping [4].

We have investigated whether replacing conventional karyotyping by SNP array analysis in cases of foetal ultrasound abnormalities would increase the diagnostic yield and speed of prenatal diagnosis in clinical practice.

In order to implement SNP array analysis we first studied its limitations. Array technology cannot detect true balanced chromosome abnormalities and non-euchromatic small supernumerary marker chromosomes (sSMC). Since these sSMCs are assumed to be benign in absence of uniparental disomy (UPD), detectable with SNP arrays [5], we further concentrated on balanced abnormalities. It is known that the prevalence of *de novo *balanced abnormalities in prenatal cohorts is low. 66 cases of apparently *de novo *balanced abnormalities were found in a cohort of 76952 samples by Warburton 1984 [6]. We have investigated the prevalence of a *de novo *reciprocal apparently balanced translocations in the population tested in our laboratory. In 1999-2010, of the total 23,357 cases, 3076 were karyotyped because of (an) ultrasound abnormality(ies). Only 2/3076 foetuses had a *de novo *apparently balanced reciprocal translocation (which is concordant to the data presented by Warburton 1984 [6]). These 2 cases were retrospectively tested with a HumanCytoSNP-12 array (HCS, 150 kb resolution). The first case (foetus with single umbilical artery, increased NT and a small stomach), who had a *de novo *apparently balanced t(1;6)(p31;q15) appeared to have a 3,8 Mb deletion at 1p31 and additionally a 3,1 Mb duplication in the 22q11 DiGeorge critical region. The second case (foetus with choroid plexus cysts and gastroschisis), who had an apparently balanced *de novo *t(9;20)(p12;q13.1), appeared to be truly balanced, when tested with HCS. Based on these data one can assume that the risk of a true balanced reciprocal translocation in foetuses with an ultrasound anomaly is very low: ~1:3000.

Low level mosaicism was another of our concerns in considering a SNP array as a primary diagnostic tool. Traditional karyotyping can exclude 26% mosaicism with 95% confidence when 10 cells are investigated, which is common practice in the Netherlands [7]. Recently, it was shown that Illumina SNP array can detect 10% of abnormal cells or even less if mosaicism involves the introduction of a new haplotype [8].

Although CGH array has also been successfully implemented into prenatal diagnosis [9], as we described before [4], we have chosen Illumina SNP array mainly because it requires only 50 ng DNA, long culturing can be avoided and rapid results can be provided within 72 hours.

From May 2009 till June 2011 723 cases of foetal ultrasound abnormalities were referred to our laboratory for prenatal cytogenetic testing. RAD (rapid aneuploidy detection) by using FISH, MLPA or QF-PCR was performed on all cases of ultrasound abnormalities. In 143 of 723 (19,7%) cases trisomy 13, 18, 21 or sex-chromosomal aneuploidy was found. In 12 of 723 (1,6%) triploidy was detected. After excluding the most common aneuploidies and triploidy by RAD the gynaecologists referred the patients to a clinical geneticist for pre-test genetic counselling and genomic array testing. After pre-test counselling (performed as described before [4]) clinical geneticists selected 207 cases for high resolution SNP array testing (including the 61 pilot cases that were previously published [4]).

In the first cases, a genomic array was requested after the karyotype appeared to be normal, later on both array and karyotyping were simultaneously performed to compare the performance and reporting time. Since September 2010 we perform array testing as a stand-alone test.

Foetal and parental DNA were simultaneously tested to exclude familial variations. The parental DNA was not always necessary to interpret the foetal array data, but to avoid the subsequent testing and speed up the analysis it was performed simultaneously to the foetal array. The array profiles were analyzed in UCSC built Hg18 (Human Mar. 2006 (NCBI36/hg18) Assembly) by using Nexus Copy Number 5.0 software (BioDiscovery) [4].

After excluding the most common trisomies and triploidy by RAD 207 cases were tested with HCS. In 24/207 (11,6%) foetuses a clinically relevant genetic abnormality was detected by genomic array (Additional file 1). A CNV was classified as clinically relevant if it was associated with a known disease or genetic syndrome or associated with an increased risk for such a disease or genetic syndrome. In 8/207 (3,7%) cases microscopically detectable abnormality was found. Submicroscopic abnormalities were found in 16/207 (7,7%) cases with foetal ultrasound abnormalities. These results are concordant to the data reported before (9% (10/106) [10], 10% (5/50) [11], 8.2% (4/49) [12]).

The use of SNP array technology instead of karyotyping increased the diagnostic yield from ~25% (19,7% common aneuploidies, 1,6% triploidy, 3,9% chromosomal abnormalities that would have been detected by karyotyping (8/207)) to ~33% (19,7% common aneuploidies, 1,6% triploidy and 11,6% all abnormalities detected by genomic array). However, the cases presented here, were highly selected (by both gynaecologists and clinical geneticists) and the percentage could change if the array testing is offered for all foetuses with ultrasound abnormalities. The reporting time was shortened from 2-3 weeks (karyotyping in AF in our center) to ≤ 1-2 weeks (array request on uncultured AF).

Only 8/16 submicroscopic abnormalities were larger than 1 Mb. Therefore, we believe that array testing with the resolution higher than 1 Mb is required for cases with foetal ultrasound abnormalities.

In 5 cases (cases 13-16 and 20 - Additional file 1) an unexpected abnormality was found. A CNV associated with an increased risk for a health condition was found. These CNVs are probably not related to the ultrasound abnormality found in the foetus and may not be sufficient to cause a phenotype. However it is known that these genomic regions have been hypothesized to be susceptibility loci. Incomplete penetrance or variable expressivity might be the explanation of the phenotype variability. The abnormal phenotype might also be caused by co-existence of an additional genetic, epigenetic, or environmental factor [13]. In such cases the correlation with the prenatal phenotype is extremely difficult as no large patients' groups with such CNVs were prenatally studied or described. Such finding could be problematic, however in our department the possibility of finding such risk factors is discussed with the patients during the pre-test counselling [4]. Whether information about risk factors was released to the patient depends on her own choice after pre-test counselling.

In 32% of our cases an unclassified variant (UV) was found. A copy number variant (CNV) was classified as an UV if it was not associated with a known human abnormal phenotype and was not seen in more than 3 healthy individuals in DGV http://projects.tcag.ca/variation/, CHOP database (http://cnv.chop.edu) or in our local databases. About 85% of these unclassified variants were inherited from a healthy parent and therefore considered to be a (private) familial polymorphism. According to our policy, results of unknown clinical significance were not revealed to the parents (during the pre-test counselling the patient consent was obtained and the consequences were discussed).

After evaluation of the advantages and disadvantages of the SNP array technology, its validation in our laboratory [4] and a year experience, we decided to replace karyotyping by a HumanCytoSNP-12 array (HCS) in cases of foetal ultrasound abnormalities for several reasons:

• HCS detects all clinically relevant unbalanced chromosome abnormalities also detected by karyotyping (including triploidy)

• HCS has a 25-50× higher resolution than karyotyping and therefore allows genome wide screening for microdeletions and microduplications.

• It can be employed on uncultured tissue (only 50 ng DNA is required)

• It is faster than karyotyping (in most cases results can be achieved within one week)

• The risk of undetected low level mosaicism is likely to be smaller than with conventional karyotyping [8,14], which is supported by our case 7 (Additional file 1: mosaic trisomy 8 (~10%) that has been missed by karyotyping).

SNP array analysis is now the preferred cytogenetic technique in cases of foetal ultrasound abnormalities after exclusion of the most common aneuploidies and triploidy. It has already been shown that the diagnostic yield can be increased by 3,6% in samples with normal karyotype regardless of the indication [15]. Since karyotyping would have missed 66% (16/24) of genomic abnormalities in our cohort, we propose to perform genomic high resolution array testing assisted by pre-test counselling as a primary prenatal diagnostic test after amniocentesis in cases of foetal ultrasound abnormalities.

## Competing interests

The authors declare that they have no competing interests.

## Authors' contributions

MIS coordinated the study, wrote the paper and studied the literature. MB performed the microarray analyses. TCB and MK did the ultrasound examination and sampling. GOO, LCPG, KEMD, RO, IMBHvdL and MJ did the genetic counselling of the parents (pre-test and post-test counselling), coordinated by R-JHG. MIS, R-JHG and DVO were responsible for the final molecular cytogenetic diagnoses and reports. All authors read and approved the manuscript.

## Supplementary Material

Additional file 1 **Table 1**. Summary of clinical significant abnormalities detected by SNP array in 24 of 207 cases. Click here for file
