# Supplementary material for: Genomic SNP array as a gold standard for prenatal diagnosis of foetal ultrasound abnormalities
Source: Mol Cytogenet. 2012 Mar 13;5:14. doi: 10.1186/1755-8166-5-14 (PMC3328283; doi:10.1186/1755-8166-5-14)
Supplement: Additional file 1 — Table 1. Summary of clinical significant abnormalities detected by SNP array in 24 of 207 cases. [file 1755-8166-5-14-S1.DOC]

*Table 1. Summary of clinical significant abnormalities detected by SNP array in 24 of 207 cases.*

| Nr | **Indication for prenatal diagnosis** | **Wk** | **M** | **Abnormality** | **Size of abnormality** | **Clinical significance** | **Karyo** |
| --- | --- | --- | --- | --- | --- | --- | --- |
| 1 | IUGR and single umbilical artery | 20.4 | uAF | arr X(0-5,296,139)x1,X(5,296,139-101,051,467)x1~2,X(101,051,467-154,913,754)x1 in a female patient | Loss of one chrX and a ring chrX - size 50Mb | Foetal karyotype: 45,X/46,X,r(X)(p22.32q22.1) (Turner syndrome) | D |
| 2 | megacystis and hydronephrosis | 15.4 | uAF | arr Xq26.3-q28(137,692,967-148,624,642)x0 | 10,9Mb loss on chrXq | interstitial deletion in band Xq26.3-q28 in a male foetus | D |
| 3 | alobar holoprosencephaly | 20.6 | uAF | arr 2p21(44,832,087-45,031,676)x1 dn | 0,2Mb loss on chr2p | Partial deletion of *SIX3* (2p21) associated with holoprosencephaly | ND |
| 4 | septated hygroma colli, and increased NT (9.1 mm) | 12.3 | LTC | arr 3p26.3p26.1(1-6,164,489)x1,19q13.12q13.43(41,151,826-63,798,055)x3 | 6,1Mb loss on chr3p and 22,6Mb gain on chr19q | unbalanced translocation: der(3)t(3;19)(p26.1;q13.12) | D |
| 5 | cleft lip | 21.2 | uAF | arr 4p16(38,283-8,321,040)x1 | 8,3Mb loss on chr4p | microdeletion 4p16 (Wolf-Hirschhorn syndrome) | ND |
| 6 | hydrocephaly | 21.4 | uAF | arr 7p14.1(42,070,719-42,206,852)x1 mat | 0,13Mb loss on chr7p | Partial deletion of *GLI3* (7p14.1) associated with Greig Cephalopolysyndactyly Syndrome | ND |
| 7 | agenesis of the corpus callosum, ventriculomegaly, IUFD | 30.3 | uAF | arr 8p23.3-q24.3(166,818-146,263,891)x2~3 | Gain of one chr8 | mosaic trisomy 8 (~10%) | ND* |
| 8 | club feet, bilateral pyelectasy | 20.2 | uAF | arr 9q34.3(137,720,637-138,083,235)x3, 9q34.3(138,083,235-140,273,252)x1 | 0,36Mb gain on chr9q and 2,2Mb loss on chr9q | terminal deletion 9q34.3 (9q34 deletion syndrome) | ND |
| 9 | IUGR, cerebral cyst, possible heart defect, echogenic bowel | 26.4 | uAF | arr 10p15.3p12.31(0-22,501,758)x1,18p11.32(0-1,236,305)x3 | 22,5Mb loss on chr10p and 1,2Mb gain on chr18p | unbalanced translocation: der(10)t(10;18)(p12.31;p11.32) dn | D |
| 10 | IUGR, abnormal thumb, cardiomyopathy (array performed on foetal fibroblasts after TOP) | 23.1 | cFIB | arr 12p13.33(61880-2980124)x1, 19p13.3p13.2(218039-7499589)x3 | 2,9Mb loss on chr 12p and 7,2Mb gain on chr 19p | unbalanced translocation: der(12)t(12;19)(p13.33;p13.2) | ND |
| 11 | semilobar holoprosencephaly, unilateral club foot, dilated right atrium without structural abnormalities of the heart | 20.5 | uAF | arr 13q31.1q34(86318347-114125098)x1 | 27,8Mb loss on chr13q | 13q31.1q34 recurrent deletion (13q- syndrome) | D |
| 12 | IUGR, polyhydramnion, dilated stomach | 33.0 | uAF | arr 14q32.13q32.31(94,793,216-100,812,042)x1 dn | 6,0Mb loss on chr14q | 14q32 recurrent deletion | D |
| 13* | increased NT (5.5 mm) | 13.1 | LTC | arr 15q11.2(19,837,058-  20,773,130)x1 | 0,9Mb loss on chr15q | recurrent deletion in 15q11.2 (NIPA2) - a risk factor for developmental delay, behavioural problems and epilepsy | ND |
| 14 | mild ventriculomegaly, suspected atrial septal defect (ASD), pericardial effusion, large foramen ovale | 20.6 | uAF | arr 15q11.2(20,305,686-20,851,614)x1 mat | 0,5 Mb loss on chr15q | recurrent deletion in 15q11.2 (NIPA2) - a risk factor for developmental delay, behavioural problems and epilepsy | ND |
| 15 | encephalocele, hydrothorax, eventration of diaphragm | 21.5 | uAF | arr 16p13.11p13.12(14,679,290-16,210,889)x3 | 1,5Mb gain on chr16p | dup(16)(p13.11p13.12) recurrent microduplication (neurocognitive disorder susceptibility locus) | ND |
| 16 | ventriculomegaly, short limbs, cardiomegaly, possible trigonocephaly | 21.1 | uAF | arr 16p11.2(28,240,364-29,297,075)x1 | 1,0Mb loss on chr16p | recurrent deletion in band 16p11.2 associated with developmental delay and obesity | ND |
| 17 | Increased NT (5 mm), abnormal first trimester screening (1:3), AMA: 39 years | 13.3 | LTC | arr 16q22.3q24.1(69,873,349-84,634,082)x1 | 14,7Mb loss on chr16q | 16q deletion | D |
| 18* | bowed femur and AMA: 40 years | 20.2 | uAF | arr 17q24.3(65,401,023-68,139,025)x1 | 2,7Mb loss on chr17q | deletion of 17q24.3: associated with campomelic dysplasia | ND |
| 19 | tetralogy of Fallot | 21.1 | uAF | arr 20p13p11.21(0-23,107,452)x3,21q22.3(45,886,003-46,944,323)x1 | 23,1Mb gain on chr20p and 1,0Mb loss on chr21q | Unbalanced translocation der(21)t(20;21)(p11.21;q11.3) | D |
| 20 | IUGR, echogenic bowel | 21.1 | uAF | arr 22q11.21(17,233,190-17,392,385)x1 pat | 0,15Mb loss on chr22q | 22q11 deletion (*PRODH*, *DGR6*)  *PRODH* testing required. Increased risk for mental retardation, autism or schizophrenia | ND |
| 21* | unilateral clubfoot, plexus choroïdeus cysts, increased NT, echogenic intracardiac focus | 21.3 | uAF | arr 22q11.21(19,367,716-19,959,004)x1mat | 0,6Mb loss on chr22q | 22q11 recurrent microdeletion (in DiGeorge/Shprintzen region of chromosome 22q11) | ND |
| 22 | VSD, hypoplastic right heart, truncus arteriosus, pulmonary valve atresia, tricuspid valve atresia | 20.0 | uAF | arr 22q11.21(18,875,330-19,959,004)x1 pat | 1,0Mb loss on chr22q | 22q11 recurrent microdeletion (in DiGeorge/Shprintzen region of chromosome 22q11) | ND |
| 23* | foetus 1: extreme IUGR, pericardial effusion, SUA, echogenic bowel | 17.5 | uAF | arr 22q11(17,249,767- 19,313,562)x1 dn | 2,0Mb loss on chr22q | 22q11 recurrent microdeletion (in DiGeorge/Shprintzen region of chromosome 22q11) | ND |
| 24 | VSD, trunctus arteriosus, plexus choroïdeus cysts | 20.6 | uAF | arr 22q11.21(17,031,504-19,791,286)x1 dn | 2,7Mb loss on chr22q | 22q11 recurrent microdeletion (in DiGeorge/Shprintzen region of chromosome 22q11) | ND |

Physical positions of the breakpoints utilize the hg18 build of the human genome sequence.

*4 cases that were published previously by Srebniak et al. 2011

AMA – Advanced Maternal Age

cFIB – Cultured Fibroblasts (sampled after TOP)

Chr – Chromosome

D –Abnormality detectable by routine karyotyping, but not fully characterised by karyotyping, since a molecular technique was necessary for assessing the breakpoints and/or translocation partner. In such cases, before introduction of array testing, multiple FISH/MLPA tests were routinely used to characterise chromosome imbalances.

dn – De novo

IUFD – IntraUterine Foetal Death

IUGR – IntraUterine Growth Retardation

LTC – Long Term Culture Chorionic Villi

M – Material

mat – Maternally inherited

ND – Abnormality not detectable during routine karyotyping

ND* - Abnormality not detected during routine karyotyping due to tissue specific mosaicism

NT – Nuchal Translucency

Karyo - Possibility to detect by conventional karyotyping

pat – paternally inherited

SUA – Single Umbilical Artery

TOP – Termination Of Pregnancy

uAF – Uncultured Amniotic Fluid

VSD - Ventricular Septal Defect

Wk - Weeks of gestation
